# Soluble TRAIL Concentration in Serum Is Elevated in People with Hypercholesterolemia

**DOI:** 10.1371/journal.pone.0144015

**Published:** 2015-12-03

**Authors:** Wen Cheng, Fangfang Liu, Zhe Wang, Yun Zhang, Yu-Xia Zhao, Qunye Zhang, Fan Jiang

**Affiliations:** 1 Key Laboratory of Cardiovascular Remodeling and Function Research, Qilu Hospital, Shandong University, 107 Wen Hua Xi Road, Jinan, 250012, Shandong Province, China; 2 Department of Endocrinology, Shandong Provincial Hospital, Shandong University, Shandong Province, China; 3 Department of Traditional Chinese Medicine, Qilu Hospital, 107 Wen Hua Xi Road, Jinan, 250012, Shandong Province, China; 4 Department of Pathophysiology, School of Medicine, Shandong University, 44 Wen Hua Xi Road, Jinan, 250012, Shandong Province, China; Osaka University Graduate School of Medicine, JAPAN

## Abstract

Tumor necrosis factor-related apoptosis-inducing ligand (TRAIL) is a multi-functional cytokine, which is involved in the pathophysiological processes of cardiovascular and metabolic diseases. Previously, we demonstrated that TRAIL stimulated lipid uptake and foam cell formation in macrophages *in vitro*. Several clinical studies have suggested that the serum concentration of TRAIL may be increased in humans with elevated blood cholesterol; however, the current data appear to be inconclusive in this regard. In the present study, we examined the relationships between the serum TRAIL concentration and cholesterol levels in 352 generally healthy subjects undergoing the routine annual health check. We showed that there were significant correlations between TRAIL concentration and levels of total and low-density lipoprotein cholesterols. The level of TRAIL was significantly elevated in subjects with hypercholesterolemia, although this relationship might be also associated with changes of other metabolic factors. Moreover, we showed that the level of blood cholesterol was significantly higher in subjects in the upper quartile of serum TRAIL. In conclusion, our data demonstrate that the serum TRAIL concentration is elevated in people with hypercholesterolemia.

## Introduction

Tumor necrosis factor-related apoptosis-inducing ligand (TRAIL) is a multi-functional cytokine, which has potent anti-tumor actions, likely via inducing cell apoptosis [1–3]. In addition, TRAIL is also involved in modulating pathophysiological processes of cardiovascular, metabolic and autoimmune diseases [4–6]. TRAIL is expressed as a transmembrane protein, which can be further cleaved by cysteine proteinases at the cell surface, generating the soluble form of TRAIL (sTRAIL). Soluble TRAIL retains the biological activities of the full length ligand [7]. A major source of sTRAIL in the body is the activated monocytes and neutrophils [2, 8]. Several clinical studies have suggested that the serum concentration of sTRAIL may be altered in heart, vascular, kidney and autoimmune diseases [9–12].

Experimental studies have indicated that TRAIL may have important roles in the pathogenesis of metabolic disorders, including obesity, diabetes and hypercholesterolemia. For example, in apolipoprotein E-deficient mice, compound deletion of the TRAIL gene resulted in increases in body weight, hyperglycemia, insulin resistance and hypercholesterolemia [13]. Also, in models of type 1 diabetes, blocking the TRAIL activity accelerated the development of hyperglycemia [14], whereas systemic overexpression of TRAIL significantly reduced the blood glucose concentration [15]. These results suggest that an elevated TRAIL level may have favorable effects on lipid and glucose metabolism under pathological conditions.

In contrast to these animal experiments, results from clinical studies on the relationship between circulating sTRAIL and metabolic parameters appear to be inconclusive. It has been shown that the serum concentration of sTRAIL is decreased in patients with diabetes mellitus as compared to healthy controls [16, 17]. However, such an association was not found in other studies [18]. Similar to this scenario, some authors reported that a higher concentration of sTRAIL was associated with increased body adiposity [19, 20], but others found no significant changes in the sTRAIL level [21]. Moreover, in a study in healthy postmenopausal women, Choi et al. reported that the serum sTRAIL concentration was significantly higher in people in the upper quartile of the blood low density lipoprotein cholesterol (LDL-C) level [19]. In another study in patients with metabolic syndromes, however, the authors did not find significant differences in TC or LDL-C between individuals with lower and higher sTRAIL concentrations [20].

In our previous study, we found that treatment with exogenous TRAIL stimulated uptake of oxidized LDL and promoted foam cell formation in both murine and human macrophages [22]. Moreover, another recent study revealed that patients with familial hypercholesterolemia were associated with an increased gene expression of TRAIL and a pro-inflammatory phenotype in the peripheral blood mononuclear cells, even on continuous statin treatments [23]. Based on these findings, we propose that it is important to further clarify the relationship between sTRAIL and hypercholesterolemia, in order to precisely define the role of TRAIL in the pathogenesis of human atherosclerosis. Therefore, in the present study, we measured serum sTRAIL concentrations in a cohort of generally healthy adults undergoing their routine annual health check, in order to confirm whether sTRAIL was increased in individuals with elevated blood cholesterol.

## Methods

### Ethical statements

This study was approved by the Institutional Human Ethics Committee of Qilu Hospital (Approval Number: KYLL-2014(KS)-003). Written informed consents were provided by all of the participants before start of the study. The study was conducted according to the principles expressed in the Declaration of Helsinki.

### Study subjects

A total of 352 subjects were recruited sequentially at the hospital’s Health Check Centre. These subjects were generally normal individuals registered to undertake their routine annual health check, but not outpatients consulting for specific disorders. A brief checklist was given to each subject and individuals with a known history of diabetes mellitus, autoimmune disease, inflammatory disease and/or coronary heart disease were excluded. There were no restrictions on other conditions such as age and sex.

### Sample processing and measurement of sTRAIL with ELISA

Venous blood of 0.5 ml of was obtained after overnight fasting from each subject. Serum samples were prepared by centrifugation at 2,000 g for 15 minutes. Samples were stored at -80°C for later use. The serum concentration of sTRAIL was measured with commercial ELISA kits purchased from various suppliers. The following kits were used: Human TRAIL/TNFSF10 Quantikine ELISA Kit (Catalogue No. DTRL00) from R&D Systems (Minneapolis, MN, USA), Human TRAIL ELISA Kit (SEA139Hu) from Uscn Life Science Inc. (Wuhan, China), and Human TRAIL ELISA Kit (EK0532) from ScienCell Research Laboratories (Carlsbad, CA, USA). Assays were carried out following the manufacturer’s protocols.

### Measurement of blood biochemical parameters and data collection

The blood biochemical tests were performed in the Central Clinical Laboratory of the hospital following standardized procedures. The blood samples were tested with an automated Cobas-8000 modular analyzer (Roche Diagnostics, Indianapolis, USA). The reagent kits for triglycerides and total cholesterol were from Roche Diagnostics GmbH (Holzheim, Germany). The reagent kits for LDL-C and high-density lipoprotein cholesterol (HDL-C) were Biosinew (Sichuan, China). Other physiological data were obtained from the participants’ health check reports.

### Statistical analysis

Data are presented as mean ± standard error of the mean (SEM). Data analysis was performed using GraphPad Prism. The mean data between two groups were analyzed with Mann-Whitney test or unpaired *t*-test. Spearman's correlation analysis was used to test the correlation between two parameters. A value of *P* < 0.05 was considered as statistically significant. Categorical data were examined with Chi-square test. All statistical tests were 2-sided.

## Results

**Fig 1:**
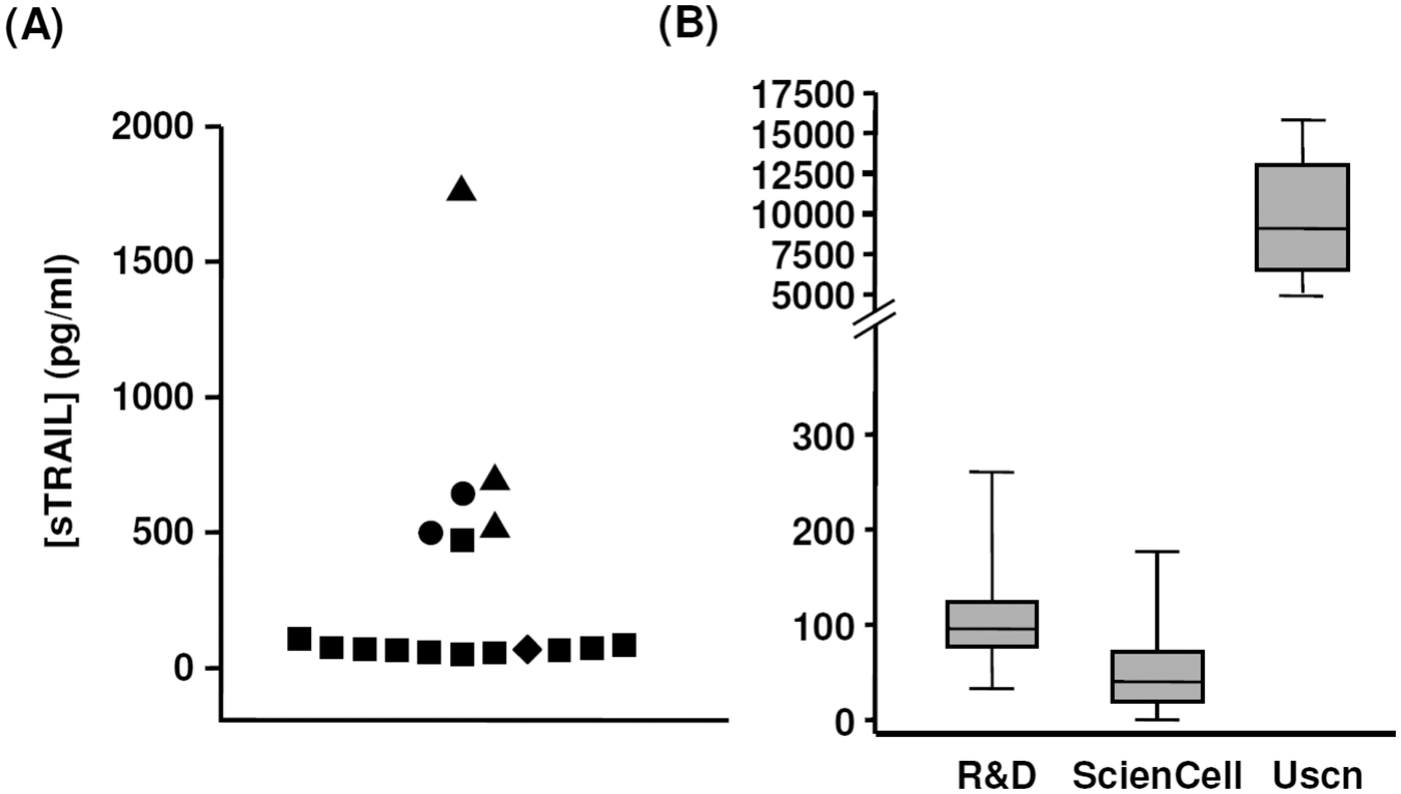
The reported values of the sTRAIL concentration in human blood in literature were highly variable, which might be related to the different assay kits used. (A) Dot plot showing the distribution of the mean sTRAIL concentrations (in pg/ml) in healthy controls reported in 17 published articles. ELISA assay kits from different suppliers were indicated by different symbol shapes. (B) Results obtained in the present study from the same set of 16 samples using ELISA kits from three different suppliers.

By reviewing the literature, we found that the reported values of the sTRAIL concentration in human blood were highly variable. We identified 17 articles that included healthy control groups and reported the absolute quantification values of sTRAIL [9, 11, 19, 21, 24–36]. The mean values of the sTRAIL concentration (in pg/ml) in healthy controls ranged from 47.3 to 1751 (median 71, mean 310.1, SEM 106.5). In these studies, ELISA kits from four different suppliers were used, and it appeared that the variability of the results might be related to the different kits used (Fig 1A). Therefore, we first tested whether different ELISA kits could generate variable results. We tested a set of 16 samples with three different commercial kits. As shown in Fig 1B, while two kits produced roughly comparable data, the third kit produced different results. Hence in the following experiments, we chose the kit from R&D Systems, which was more frequently used in previous studies. We tested the variability of the assay, and determined that the intra- and inter-assay coefficient of variation was 5.6% and 9.8% respectively. However, it is noted that the different absolute sTRAIL values as determined by different kits are of little impacts on the conclusions from these studies.

**Fig 2:**
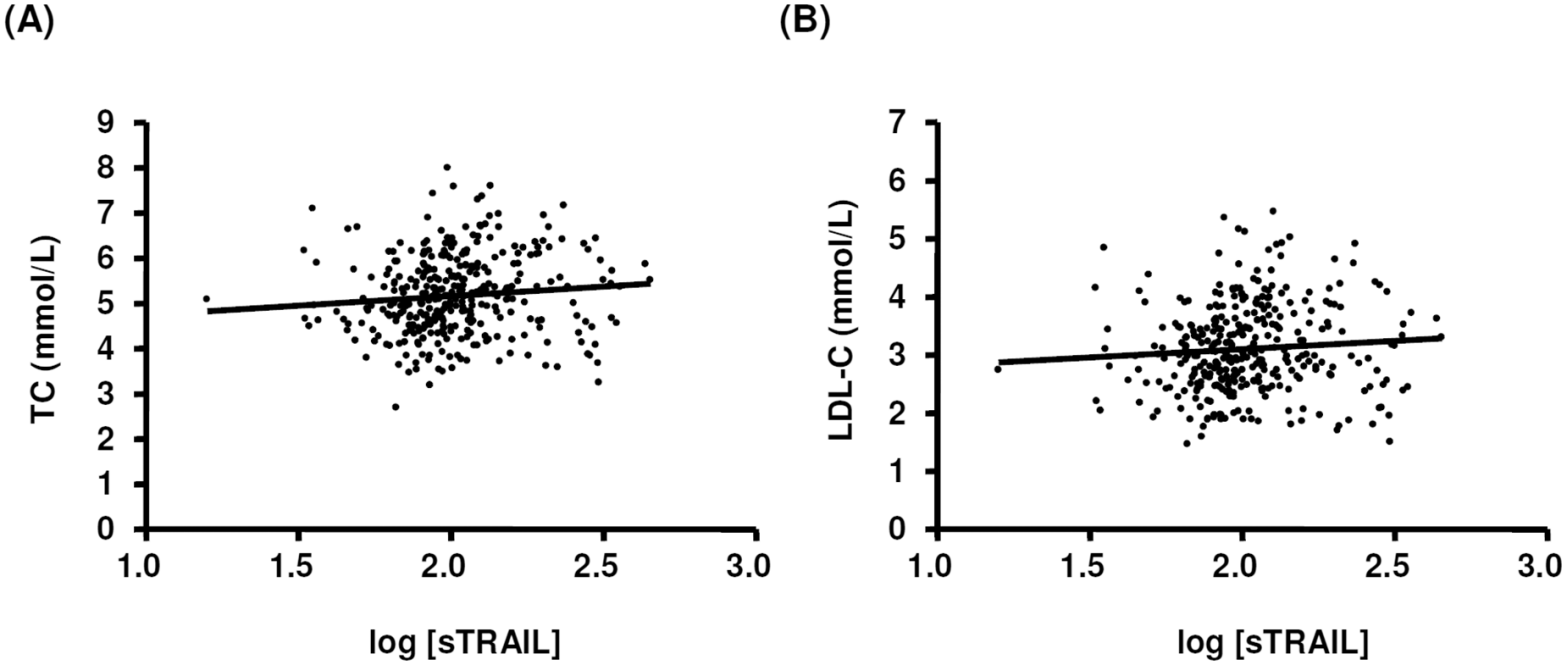
Dot plots showing correlations between sTRAIL and (A) total cholesterol (TC) or (B) low-density lipoprotein cholesterol (LDL-C) in the entire cohort. The sTRAIL values were log10 transformed. Spearman's correlation was used and the statistical results were given in Table 3.

The general demographic characteristics of the 352 subjects were summarized in Table 1. The age of the subjects ranged from 30 to 68 years. In this cohort, ~15% had a blood pressure that exceeded the normal value. In about half of the subjects, the total cholesterol level was higher than the normal value (5.2 mmol/L). According to the standard commonly used within China, > 45% of these subjects were overweight (body mass index, BMI > 24) or obese (BMI > 28). Comparisons of the physiological and blood biochemical parameters between individuals with normal and elevated (> 5.2 mmol/L) TC were given in Table 2. The mean sTRAIL concentration of the 352 subjects was 117.3 ± 3.5 pg/ml. The level of sTRAIL was significantly higher in females (133.2 ± 7.3) than males (108.7 ± 3.6) (*P* < 0.05). Correlation analysis revealed that there were weak but significant correlations between sTRAIL and TC and LDL-C (Table 3 and Fig 2). In contrast to previous studies [20], we detected negative correlations between sTRAIL and BMI or body weight, for which the reason of the discrepancy was unclear. The level of sTRAIL did not correlate with age, blood pressure, or serum levels of triglycerides and HDL-C (Table 3).

**Table 1:** General physiological and biochemical characteristics of 352 subjects.

Age (mean, SEM, range)	45.7, 0.37, 30–68
Sex (% male)	64.8
SBP > 140 and/or DBP > 90 mmHg (%)	15.6
TC > 5.2 mmol/L (%)	47.2
LDL-C > 3.1 mmol/L (%)	46.6
Overweight (BMI > 24) (%)	37.8
Obesity (BMI > 28) (%)	9.1

SBP, systolic blood pressure; DBP, diastolic blood pressure; TC, total cholesterol; LDL-C, low-density lipoprotein cholesterol; BMI, body mass index.

**Table 2:** Comparisons of physiological and biochemical parameters between individuals with normal and elevated TC.

	TC (mmol/L)		
	< 5.2 (n = 185)	> 5.2 (n = 166)	*P*
Age (median years)	43	46	0.0041
Sex (% male)	59.1	71.1	0.0192
SBP (mmHg)	120 ± 1.2	127 ± 1.3	0.0002
DBP (mmHg)	78.7 ± 0.80	82.8 ± 0.91	0.0008
TC (mmol/L)	4.49 ± 0.035	5.93 ± 0.044	---
LDL-C (mmol/L)	2.58 ± 0.032	3.69 ± 0.045	<0.0001
HDL-C (mmol/L)	1.30 ± 0.023	1.36 ± 0.023	0.047
TG (mmol/L)	1.39 ± 0.066	1.86 ± 0.098	<0.0001
BW (kg)	70.5 ± 0.97	72.2 ± 0.94	0.207
Overweight (%)	56.2	62.2	0.257
Obesity (%)	15.7	17.1	0.725
Hyperglycemia (%)	0.54	1.2	0.497

SBP, systolic blood pressure; DBP, diastolic blood pressure; TC, total cholesterol; LDL-C, low-density lipoprotein cholesterol; HDL-C, high-density lipoprotein cholesterol; TG, triglycerides; BW, body weight. Hyperglycemia was defined as glucose > 6.1 mmol/L.

**Table 3:** Correlations between sTRAIL and total cholesterol with other physiological and biochemical parameters in 352 subjects.

	sTRAIL		TC	
	Spearman r	*P*	Spearman r	*P*
Age	0.020	0.716	0.164	0.002
BMI	-0.197	0.0002	0.106	0.047
TC	0.164	0.002	---	---
TG	-0.010	0.852	0.320	<0.0001
HDL-C	0.065	0.225	0.130	0.015
LDL-C	0.133	0.013	0.901	<0.0001
SBP	-0.0082	0.879	0.273	<0.0001
DBP	-0.0080	0.882	0.243	<0.0001
BW	-0.176	0.0009	0.119	0.027
sTRAIL	---	---	0.164	0.002

BMI, body mass index; TC, total cholesterol; TG, triglycerides; HDL-C, high-density lipoprotein cholesterol; LDL-C, low-density lipoprotein cholesterol; SBP, systolic blood pressure; DBP, diastolic blood pressure; BW, body weight.

**Fig 3:**
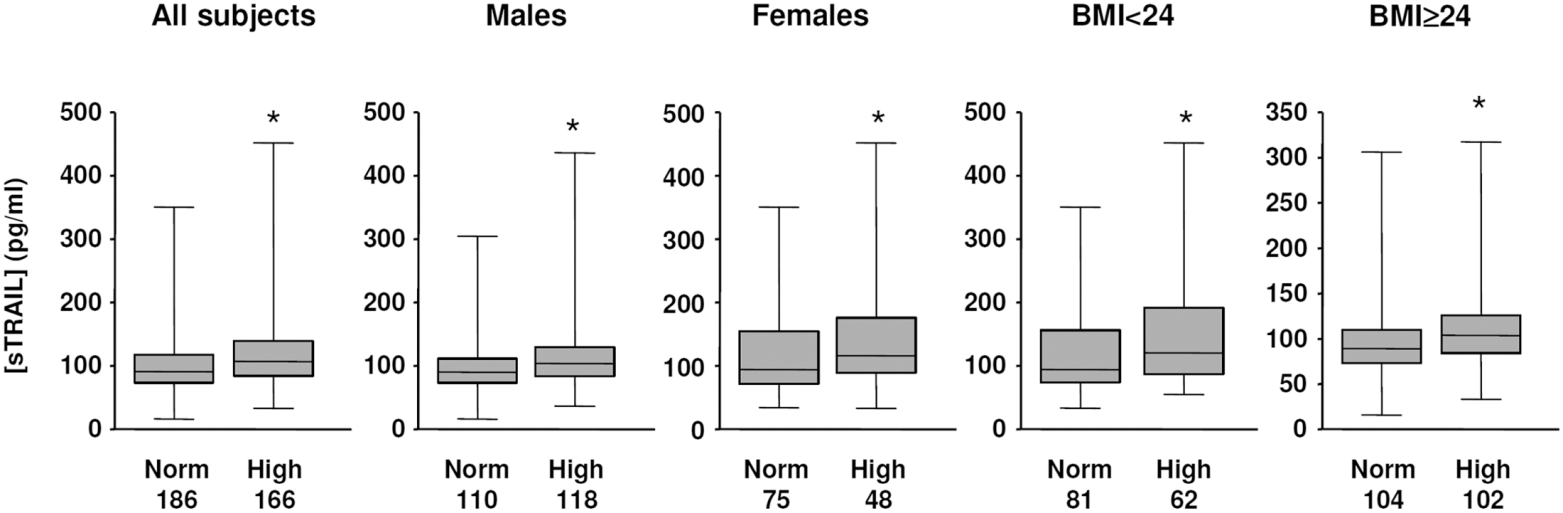
Comparison of sTRAIL concentrations in subjects with normal (Norm) and elevated (High) blood total cholesterol using 5.2 mmol/L as a cutting-off value, in the total 352 subjects and in different subject categories according to gender and body mass index (BMI). The *n* numbers included in each (sub)group were shown under the columns. * *P* < 0.05, Mann-Whitney test.

Using TC level of 5.2 mmol/L as a cutting-off value, the subjects were divided into two groups, namely those with normal (TC-Norm group) and elevated (TC-high group) blood total cholesterol levels. We demonstrated that the sTRAIL concentration was significantly higher in the TC-high group (*P* < 0.001) (Fig 3). We also tested the difference in sTRAIL concentration using TC of 5.7 mmol/L as the cutting-off value. We confirmed that the sTRAIL concentration was still significantly higher in the TC-high group (126.8 ± 6.7) than the TC-Norm group (113.6 ± 4.1) (*P* < 0.005). To verify whether such a relationship between sTRAIL and TC held in different subgroups of the subjects, we first made comparisons in males and females separately. As shown in Fig 3, the mean sTRAIL concentration in males was significantly higher in TC-high than TC-Norm individuals. A similar difference of sTRAIL concentration was also present in females. Then we divided the subjects into two groups according to their BMI, namely < 24 (normal bodyweight) and ≥ 24 (overweight/obese). In these two subgroups, significant differences in mean sTRAIL concentration between TC-high and TC-Norm individuals were consistently detected (Fig 3). Moreover, a significantly higher sTRAIL concentration was also observed in TC-high individuals in different age bands. In younger people (< 45 years), sTRAIL concentrations were 116.2 ± 6.6 pg/ml in TC-high versus 109.2 ± 6.1 in TC-Norm individuals (*P* = 0.0293). In older people (≥ 45 years), sTRAIL concentrations were 134.5 ± 8.1 in TC-high versus 109.1 ± 6.7 in TC-Norm individuals (*P* = 0.0036).

**Fig 4:**
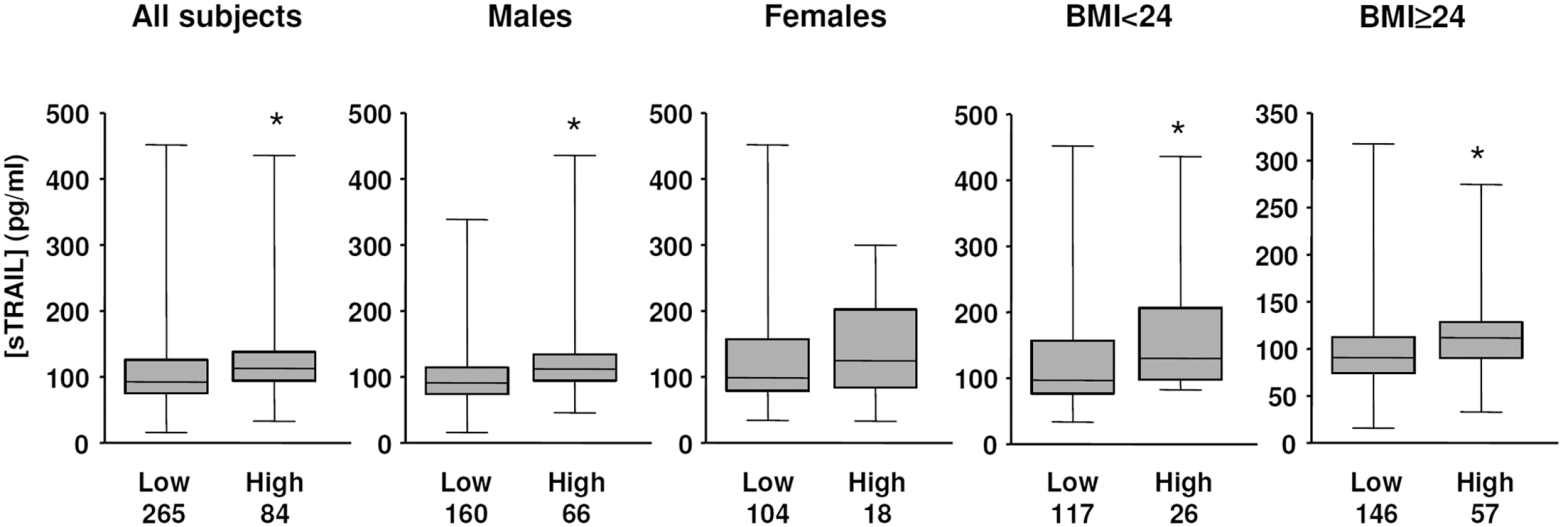
Comparison of sTRAIL concentrations in subjects with lower and higher blood LDL-cholesterol using 3.6 mmol/L as a cutting-off value, in the total 352 subjects and in different subject categories according to gender and body mass index (BMI). The *n* numbers included in each (sub)group were shown under the columns. * *P* < 0.05, Mann-Whitney test.

Next we examined whether the sTRAIL concentration was changed with the blood LDL-C level. Using a cutting-off value of 3.1 mmol/L of LDL-C, we did not detect a significant difference between LDL-low versus LDL-high subjects (113.8 ± 4.6 and 121.8 ± 5.5 pg/ml respectively, *P* = 0.073). However, using a cutting-off value of 3.6 mmol/L, we found that the sTRAIL concentration was significantly higher in the LDL-high group (*P* < 0.0005) (Fig 4). We further confirmed that the mean sTRAIL concentration in males was significantly higher in the LDL-high than LDL-low individuals (Fig 4). However, the difference in females was not significant (Fig 4). In relation to the body adiposity, we found that in both of the normal bodyweight group and the overweight/obese group, the mean sTRAIL concentrations were all significantly higher in LDL-high individuals (Fig 4). In younger people, sTRAIL concentrations were 123.3 ± 7.8 in LDL-high versus 109.2 ± 5.3 in LDL-low individuals (*P* = 0.0013). In older people, sTRAIL concentrations were 132.3 ± 14.3 in LDL-high versus 118.1 ± 6.0 in LDL-low individuals, of which the difference was not significant (*P* = 0.1851). We could not totally exclude the possibility that the lack of significance was due to the relatively small size of these subgroups.

Finally, we compared various physiological and biochemical parameters between subjects in the lower and upper quartiles of the sTRAIL concentration. The mean age was not different between the two quartiles. TC and LDL-C levels in the upper quartile were significantly higher than those in the lower quartile (4.89 ± 0.08 versus 5.28 ± 0.10 for TC, *P* = 0.0056; 2.89 ± 0.07 versus 3.15 ± 0.09 for LDL-C, *P* = 0.042), whereas there were no significant differences in triglycerides, HDL-C, SBP or DBP. BMI was also different between the two quartiles (S1 Table).

## Discussion

Here we provided data showing that in humans there were significant correlations between the serum sTRAIL concentration and levels of total and LDL cholesterol. The level of sTRAIL was significantly elevated in people whose TC and/or LDL-C were above the normal values. This kind of relationship between sTRAIL and hypercholesterolemia retains in subgroups of subjects with different age, gender and the status of adiposity (except for LDL-C in females and those ≥ 45 years of age). Moreover, we showed that the levels of TC and LDL-C were significantly higher in people in the upper quartile of sTRAIL than those in the lower quartile. Using a more generalized population, we confirmed previous findings in postmenopausal women [19], and further established the positive correlation between sTRAIL and blood cholesterol levels observed in subjects with metabolic anomalies [20]. It should be noted that, however, the increase in sTRAIL concentration in hypercholesterolemia cannot establish that sTRAIL is an independent biomarker for the metabolic disorders. Given that the change in TC is concomitant with changes in various other metabolic parameters, it is likely that the sTRAIL concentration may be also affected directly or indirectly by other metabolic factors.

The relationship between sTRAIL and hypercholesterolemia in humans seems to be in contrast to the results obtained from animal experiments, which indicate a hypolipidemic action of endogenous TRAIL in vivo [13]. The physiological and/or pathophysiological significance of the elevated sTRAIL concentration in human hypercholesterolemia is currently not understood. On one hand, evidence has shown that there is an inverse relationship between the serum sTRAIL concentration and the severity of coronary arterial disease [12, 33, 37], indicating that the increased TRAIL production may act as a compensatory mechanism that regulates the disrupted homeostasis of lipid metabolism under pathological conditions, and may prevent the development of resultant vascular lesions. On the other hand, elevated TRAIL may be a pathogenic factor that potentially contributes to hypercholesterolemia-induced vascular injuries. Indeed, there is evidence showing that increased TRAIL may cause apoptosis and inflammatory responses in vascular endothelial and/or smooth muscle cells [38]. Moreover, there is evidence showing that TRAIL may induce lipid uptake and foam cell formation in macrophages [22]. Therefore, our results suggest that further investigations on the precise roles of TRAIL in human hypercholesterolemia and atherosclerosis are warranted.

Whether males and females have different basal levels of sTRAIL is controversial based on previous results [19, 39]. In the present study, we confirmed that the basal sTRAIL concentration was significantly higher in women than men, which was consistent with the results reported by another group [39]. Previous studies also found that the sTRAIL concentration was positively correlated with indices of obesity [19, 20]. However, in this study we did not detect a significant correlation between sTRAIL and BMI. A limitation of our study was that, apart from BMI, we did not measure other obesity-related parameters such as waist circumference and fat mass. We could not exclude that BMI did not precisely reflect the status of mass and distribution of the body fat. Indeed, in another study in Caucasians, the authors also did not find a significant difference in sTRAIL levels between normal weight and obese subjects based on BMI [21].

In summary, our data have confirmed that the serum sTRAIL concentration is elevated in humans with hypercholesterolemia. Generation of sTRAIL bestows this cytokine the ability to act as a paracrine factor. Although data from animal experiments have suggested that TRAIL have beneficial effects on lipid metabolism, additional clinical evidence is required to establish whether an increased sTRAIL concentration in humans confers vascular protection, or represents a potential pathogenic factor that may further facilitate lesion formation in the presence of hypercholesterolemia, which is a most important risk factor for atherosclerosis and coronary arterial disease.

## Supporting Information

S1 Table Comparison of various physiological and biochemical parameters between subjects in the lower and upper sTRAIL quartiles (DOC)

S2 Table Data set of concentrations of sTRAIL, total cholesterol and low-density lipoprotein cholesterol (PDF)
