# Supplementary material for: Soluble TRAIL Concentration in Serum Is Elevated in People with Hypercholesterolemia
Source: PLoS One. 2015 Dec 3;10(12):e0144015. doi: 10.1371/journal.pone.0144015 (PMC4669162; doi:10.1371/journal.pone.0144015)
Supplement: S1 Table — (DOC) [file pone.0144015.s001.doc]

**Table S1**. Comparison of various physiological and biochemical parameters between subjects in the lower and upper sTRAIL quartiles

|  | **sTRAIL concentration** | |  |
| --- | --- | --- | --- |
|  | **Lower quartile** | **Upper quartile** | ***P*** |
| **Age** | 45.9 ± 0.80 | 45.4 ± 0.72 | 0.85 |
| **TC** | 4.89 ± 0.08 | 5.28 ± 0.10 | 0.0056 |
| **LDL-C** | 2.89 ± 0.07 | 3.15 ± 0.09 | 0.042 |
| **TG** | 1.62 ± 0.12 | 1.54 ± 0.12 | 0.45 |
| **HDL-C** | 1.30 ± 0.03 | 1.38 ± 0.03 | 0.060 |
| **BMI** | 25.3 ± 0.34 | 23.6 ± 0.40 | 0.0006 |
| **SBP** | 123.4 ± 1.8 | 122.6 ± 1.8 | 0.58 |
| **DBP** | 80.4 ± 1.2 | 79.7 ± 1.2 | 0.54 |

TC, total cholesterol; TG, triglycerides; HDL-C, high-density lipoprotein cholesterol; LDL-C, low-density lipoprotein cholesterol; BMI, body mass index; SBP, systolic blood pressure; DBP, diastolic blood pressure. Data are mean ± SEM. Mann-Whitney test.
