# Supplementary material for: Soluble TRAIL Concentration in Serum Is Elevated in People with Hypercholesterolemia
Source: PLoS One. 2015 Dec 3;10(12):e0144015. doi: 10.1371/journal.pone.0144015 (PMC4669162; doi:10.1371/journal.pone.0144015)
Supplement: S2 Table — (PDF) [file pone.0144015.s002.pdf]

sTRAIL	TCmmol/L	LDLmmol/L
15.9	5.08	2.74
33.2	6.16	4.15
33.4	4.65	2.2
34.4	4.49	2.04
35.4	7.09	4.84
35.7	4.95	3.1
36.4	5.89	3.43
36.9	4.62	2.8
42.5	4.8	2.56
44.8	4.63	2.97
46.0	4.39	2.74
46.2	6.63	4.09
46.4	4.53	2.17
48.3	5.74	3.9
48.8	4.17	2.51
49.6	6.68	4.38
51.3	4.55	1.92
51.8	5.1	3.14
53.1	3.79	2.02
53.4	4.88	3
53.8	4.83	2.53
55.2	5.56	3.46
55.2	4.93	3.11
55.9	4.4	2.93
56.5	4.15	2.41
58.2	4.26	2.43
60.3	4.85	2.63
60.9	5.35	3.53
61.9	4.08	2.27
62.1	5.62	3.48
62.5	4.75	2.99
63.0	5.74	3.46
63.0	6.14	3.97
63.3	4.05	2.07
63.4	4.71	2.84
63.8	4.94	3.2
65.0	5.04	2.37
65.1	6.13	3.9
65.4	4.99	3.15
65.4	4.72	2.91
65.6	5.43	3.29
65.7	5.27	3.09
65.8	5.46	3.14
66.0	5.92	3.3
66.4	2.69	1.46
66.5	4.59	2.97
67.1	5.92	3.92

67.1	4.69	3.01
67.8	3.63	1.89
68.2	5.58	3.5
68.3	4.1	2.5
68.7	6.33	3.49
68.8	4.93	2.61
69.0	4.77	
69.5	5.31	3.4
70.1	5.05	3.32
70.1	4.81	2.67
70.4	5.22	2.97
70.4	5	2.74
70.8	4.14	2.24
71.5	4.32	2.37
71.7	4.92	2.97
71.8	3.84	2.41
71.8	4.96	2.5
72.2	5.34	3.29
73.3	4.97	3.43
73.3	4.96	2.79
73.3	3.46	2.1
73.4	4.5	2.45
73.7	4.68	2.53
73.9	4.38	1.59
74.0	4.72	3.2
74.4	5.52	3.36
74.6	6.15	3.34
75.0	4.45	1.76
75.5	4.51	2.86
75.6	4.5	2.69
76.2	4.32	2.63
77.1	5.35	3.45
77.4	3.67	1.88
77.5	3.53	1.9
77.5	5.46	3.23
77.6	4.67	2.21
78.3	4.12	2.61
78.5	4.1	2.61
78.7	4.45	2.8
78.8	5.7	3.81
78.9	4.08	2.66
79.2	4.37	2.19
80.1	5	3.13
80.2	4.78	3.06
80.5	4.97	3.06
81.0	4.99	3.39
81.2	3.85	1.92
81.3	4.22	1.96

81.4	5.2	3.17
81.5	5.57	3.59
81.6	4.21	2.76
81.7	6.07	3.5
81.8	4.43	2.46
81.8	5.87	3.19
82.1	4.72	3.05
82.3	6.31	4.07
82.3	5.82	3.62
82.4	6.14	3.85
82.4	5.66	2.86
82.7	6.1	3.56
82.8	4.51	2.87
83.1	4.49	2.47
83.3	5.21	2.79
83.7	4.74	2.83
83.9	5.36	3.21
84.0	6.37	3.82
84.0	5.71	3.44
84.3	6.89	4.74
84.8	5.77	4.2
84.8	4.63	2.69
85.0	4.6	2.44
85.1	3.74	1.97
85.3	6.16	4.04
85.3	5.05	3.32
85.6	3.18	1.89
85.7	4.87	2.72
85.7	3.84	1.96
86.0	5.73	3.43
86.0	5.51	3.64
86.1	4.52	2.58
86.5	4.33	2.56
86.7	4.61	2.4
87.0	6.02	3.83
87.5	5.13	2.93
87.7	7.42	5.36
88.2	4.62	1.9
88.4	5.33	3.05
88.8	5.73	3.02
89.0	5.18	2.73
89.1	5.32	3.55
89.1	5.56	3.2
89.2	4.71	3
89.3	4.6	2.36
89.5	5.07	2.62
89.9	5.23	2.96
90.7	4.81	2.48

90.8	5.53	2.54
91.0	4.81	3.21
91.0	4.33	2.38
91.3	4.09	2.89
91.7	5.08	2.94
91.8	5.51	3.33
91.8	3.53	2.28
91.9	5.13	3.2
92.7	4.68	2.86
92.9	5.29	3.13
93.3	4.79	3.16
93.6	6.6	4.13
94.0	6.05	4.11
94.1	3.92	2.34
94.2	4.37	2.27
94.2	4.82	2.61
94.3	5.3	3.34
94.4	4.66	2.63
94.4	3.48	1.99
94.8	6.05	4
94.9	4.49	2.72
95.1	4.6	2.53
95.4	4.44	2.72
95.4	4.31	2.64
95.4	6.04	3.62
95.6	6.15	3.82
95.8	6.12	4.14
95.9	4.88	2.94
96.4	5.16	2.9
96.7	4.96	2.9
96.7	6.32	4.14
97.1	5.48	2.68
97.5	4.2	2.03
97.8	7.99	5.16
98.1	6.44	4.55
98.2	6.04	3.51
98.2	5.16	3.38
99.2	6.4	4.14
99.2	5.7	3.46
99.9	3.81	2.44
100.1	4.95	2.92
100.1	5.99	4.05
100.2	5.01	3.37
100.3	4.47	2.74
100.4	6.28	4.03
100.7	4.37	2.57
100.7	4.82	3.05
101.1	6.22	3.94

101.2	5.58	3.33
101.5	5.23	3.01
101.5	3.56	1.89
101.5	6.43	3.99
102.5	7.58	5.11
102.6	5.59	3.45
103.1	6.32	3.98
103.3	5.37	3.47
104.2	4.48	2.48
104.2	5.07	2.95
104.3	5.56	3.7
104.5	4.93	
105.8	5.95	3.45
105.8	3.84	2.02
106.3	5.36	3.1
106.4	5.6	3.44
107.6	5.25	3.04
107.8	5.81	1.89
108.4	3.75	
108.9	4.2	2.78
109.5	5.01	3.15
109.6	5.28	3.1
109.8	5.52	3.19
109.9	6.07	4.3
109.9	4.81	2.55
110.0	4.91	2.54
110.4	5.39	3.71
110.9	5.14	3
111.3	5.52	3.29
111.5	6.14	3.89
112.1	4.83	2.84
112.2	6.13	3.87
112.7	6.68	4.44
112.7	5.82	3.95
112.8	5.84	3.59
113.0	6.1	3.59
113.5	3.72	1.85
113.6	6.17	4.26
113.8	4.24	2.62
114.2	4.39	2.53
114.7	4.62	2.91
116.2	5.68	3.85
116.4	4.82	3.07
116.7	4.71	2.41
116.8	4.33	2.9
117.0	4.74	2.8
117.0	4.56	2.37
117.3	5.81	3.99

117.7	5.06	3.61
118.1	5.22	2.42
118.2	4.94	2.82
118.7	5.77	3.78
118.8	4.6	2.73
119.2	5.81	3.52
119.2	6.5	4.12
119.6	4.06	2.27
121.9	5.63	3.97
122.2	5.24	3.32
122.4	5.78	4.1
122.6	4.06	2.41
122.6	6.34	3.84
122.8	7.29	4.8
123.2	5.87	3.67
123.5	6.47	3.98
125.4	6.72	4.45
126.1	4.53	2.45
126.2	6.69	4.18
126.3	5.29	3.52
127.0	7.36	5.46
127.1	4.96	2.67
128.5	5.22	3.12
129.1	5.36	3.77
129.6	5.78	3.5
130.2	4.26	2.74
130.3	6.74	4.89
130.8	5.29	3.25
131.5	4.15	2.61
132.1	4.93	3.47
133.3	5.67	3.44
133.8	4.19	2.46
134.2	5.42	3.65
134.7	6.92	4.69
135.4	7.59	4.92
135.5	6.44	4.05
138.4	5.2	3.08
140.1	5.35	2.92
140.5	6	4.15
140.8	4.91	2.98
142.1	4.8	2.86
142.8	5.14	2.4
142.8	4.25	2.03
144.1	6.97	5.02
144.5	6.68	3.89
145.2	3.71	1.8
146.1	4.67	2.72
148.7	6.11	3.7

152.0	5.27	2.63
153.1	5.07	3.38
153.6	5.32	2.92
154.9	5.09	2.58
156.1	5.33	3.24
157.4	3.88	1.86
158.3	4.79	2.98
159.4	5.36	3.24
159.6	4.19	2.06
160.4	5.08	2.8
162.1	5.86	3.92
163.3	6.25	3
166.6	5.48	2.88
166.8	6.09	4.07
167.2	5.87	3.54
169.5	5.64	3.75
173.4	6.22	2.98
175.1	5.01	2.79
175.3	4.28	2.74
180.3	3.84	1.96
183.8	4.64	2.86
184.1	6.06	3.43
189.6	6.35	3.91
191.6	6.08	3.55
192.9	5.36	3.48
194.2	6.23	4.13
194.7	4.6	2.66
195.4	6.09	3.87
196.7	4.45	2.64
199.0	4.62	2.78
201.9	6.37	3.86
202.4	6.94	4.64
206.1	3.61	1.7
208.7	4.12	1.77
210.1	6.68	4.22
211.1	6.23	3.81
214.3	5.46	3.39
224.8	3.58	1.87
229.2	5.56	2.97
232.7	6.41	4.57
235.6	7.16	4.91
242.8	5.36	2.85
253.5	5	2.37
260.8	4.71	2.93
264.2	4.34	2.44
269.2	4.13	1.8
274.2	6.31	4.25
278.8	4.58	2.72

282.9	3.87	2.08
283.6	6.18	4.2
287.6	3.84	2.09
291.4	4.47	2.48
299.6	4.08	2.56
299.9	6.43	4.08
304.8	3.67	1.95
306.2	3.24	1.5
310.7	5.94	3.17
317.5	5.51	3.15
336.7	5.43	3.33
338.0	4.67	2.38
338.9	5.71	3.52
350.4	4.56	2.44
359.4	5.36	3.72
436.2	5.86	3.62
451.8	5.51	3.3
